# Endoscopic transection of right L3 and L4 medial branch nerves and L5 dorsal ramus: A 2-dimensional operative video

**DOI:** 10.1016/j.inpm.2022.100142

**Published:** 2022-09-24

**Authors:** Chris Burton, Reza Ehsanian, Jason Capra, W. Evan Rivers, Peter Shin

**Affiliations:** a University of New Mexico School of Medicine, USA; b NeuroMicroSpine, Pensacola, FL, USA; c Tennessee Valley Healthcare System, Veterans Administration, Nashville, TN, USA; d Department of Physical Medicine and Rehabilitation, Vanderbilt University Medical Center, USA

**Keywords:** Endoscopic rhizotomy, Endoscopic, Medial branch nerve, Dorsal rami, Lumbar zygapophyseal joint, Axial low back pain

Zygapophyseal joints in the lumbar spine are innervated by medial branches of the dorsal rami of spinal nerves and have been implicated in the generation of axial low back pain. Percutaneous radiofrequency ablation procedures target bony landmarks under fluoroscopic guidance near the anticipated location of medial branches and dorsal rami. In contrast, endoscopic visualization allows for surgical transection of the targeted nerves through either direct visualization or dissection and ablation of the tissues surrounding the expected trajectory of the nerves. Endoscopic transection of medial branches and dorsal rami has been suggested as a possible alternative to percutaneous radiofrequency lesioning although further research is needed to define indications, explore relative risks and benefits, and evaluate efficacy by directly comparing these two techniques.

In this video, we present a case of a female with chronic axial low back pain that was refractory to conservative care including physical therapy, exercise, facet steroid injections, and analgesic medications. After positive response to blocking the L3, L4 medial branch nerves and the L5 dorsal ramus, the patient underwent multiple percutaneous radiofrequency ablations, which provided progressively shorter duration of relief. With her persistent symptoms suggestive of facet-mediated lumbar back pain, endoscopic transection was offered as an alternative approach. We provide an operative video including discussion of imaging, target anatomy and operative steps for endoscopically-visualized transection of the L3 and L4 medial branch nerves and the L5 dorsal ramus on the right side [[1], [2], [3], [4]].

## Declaration of competing interest

The authors declare that they have no known competing financial interests or personal relationships that could have appeared to influence the work reported in this paper.

## References

[1] Schneider B.J., Doan L., Maes M.K. (2020). Systematic review of the effectiveness of lumbar medial branch thermal radiofrequency neurotomy, stratified for diagnostic methods and procedural technique. Pain Med.

[2] Yeung A., Gore S. (2014). Endoscopically guided foraminal and dorsal rhizotomy for chronic axial back pain based on cadaver and endoscopically visualized anatomic study. Internet J Spine Surg.

[3] Xue Y., Ding T., Wang D. (2020). Endoscopic rhizotomy for chronic lumbar zygapophysial joint pain. J Orthop Surg Res.

[4] Lau P., Mercer S., Govind J., Bogduk N. (2004). The surgical anatomy of lumbar medial branch neurotomy (facet denervation). Pain Med.

